# Cardiac magnetic resonance imaging: the future is bright

**DOI:** 10.12688/f1000research.19721.1

**Published:** 2019-09-13

**Authors:** Karthik Seetharam, Stamatios Lerakis

**Affiliations:** 1 Division of Cardiology, Icahn School of Medicine at Mount Sinai, Mount Sinai Medical Center, New York, USA

**Keywords:** T1 mapping, T2 mapping, Artificial Intelligence, Myocardial Strain

## Abstract

Over the last 15 years, cardiovascular magnetic resonance (CMR) imaging has progressively evolved to become an indispensable tool in cardiology. It is a non-invasive technique that enables objective and functional assessment of myocardial tissue. Recent innovations in magnetic resonance imaging scanner technology and parallel imaging techniques have facilitated the generation of T1 and T2 parametric mapping to explore tissue characteristics. The emergence of strain imaging has enabled cardiologists to evaluate cardiac function beyond conventional metrics. Significant progress in computer processing capabilities and cloud infrastructure has supported the growth of artificial intelligence in CMR imaging. In this review article, we describe recent advances in T1/T2 mapping, myocardial strain, and artificial intelligence in CMR imaging.

## Introduction

Cardiovascular magnetic resonance (CMR) imaging has rapidly emerged as a robust diagnostic option for evaluating a number of pathological entities in cardiology. Furthermore, CMR is the gold standard for non-invasive measurement of left ventricular and right ventricular volumes and ejection fraction ^1^. CMR enables non-invasive cardiac visualization with augmented temporal and spatial resolution along with high blood-to-tissue contrast ^2^. Moreover, it allows detailed tissue characterization which is pivotal to its ability to not only diagnose cardiovascular disease but also facilitate management and treatment. It can precisely determine myocardial and vascular injury ^2^. Recent advances in software and technological hardware have propelled the development of new methods that can substantially augment cardiovascular diagnosis, prognosis, and risk stratification ^3^. CMR permits visual assessment through a variety of approaches, which include late gadolinium enhancement (LGE), T1 mapping, and T2 mapping ^1^. Strain imaging is an emerging diagnostic modality that can serve as a prognostic imaging marker to evaluate myocardial function beyond the ejection fraction ^4^. Artificial intelligence (AI) has opened new frontiers in cardiology by leading to data-driven discoveries in CMR imaging ^5, 6^.

In this review article, we aim to assess advances in CMR evaluation of various cardiovascular conditions over the last few years. This will include T1/T2 mapping, the role of AI in CMR, and the significance of myocardial strain in CMR.

## T1 mapping

Over the last few decades, LGE has been used to detect focal areas of fibrosis in the myocardium. The presence of diffuse fibrosis has been linked to diastolic dysfunction, heart failure, and sudden death ^7^. Although it is a valuable tool, it identifies only localized areas of tissue damage, often where there is irreversible replacement fibrosis ^7^. This technique requires an area of normal myocardium as a reference point in order to highlight the areas of fibrosis and this limitation can be viewed as the Achilles’ heel of LGE. Therefore, there is substantial interest in approaches that permit identification of early fibrosis as well as more diffuse fibrosis. T1 mapping can characterize the myocardial tissue on a pixel-to-pixel basis to generate a map of T1 values. T1 mapping is able to identify earlier diffuse fibrosis and can detect subtle changes in heart muscle pathology in a non-invasive manner ^7^. In addition, T1 mapping may allow more precise quantification of an area of infarction ^1^. T1 mapping also enables extracellular volume measurement (ECV) which can also measure myocardial fibrosis in reference to left ventricular volume ^7^.

## Recent studies with T1 mapping

Involvement of the myocardium can occur in systemic light-chain amyloidosis. Although LGE can identify characteristic amyloid patterns, these are evident in later stages of the disease. Karamitsos *et al*. explored the role of non-contrast myocardial T1 mapping for recognizing cardiac involvement in 53 patients with amyloid light-chain amyloidosis and 53 control subjects ^8^. The myocardial T1 was substantially higher in the patients with amyloidosis (1140 ± 61 ms, *P* <0.0001) than in the other patients. A non-contrast myocardial T1 cutoff of 1020 ms showed an accuracy of 92% for revealing cardiac involvement in patients with amyloidosis. Furthermore, there were significant associations between myocardial T1 values and systolic and diastolic dysfunction indices.

Low-flow low-gradient aortic stenosis (LFLG AS) has high operative risk for surgical intervention and poor prognosis with medical management. However, there is a paucity of data on the degree of fibrosis in these patients. Dobutamine stress echocardiography is traditionally used to evaluate the left ventricular flow reserve (FR) to confirm the severity of AS, although there are conflicting data on FR mortality predictive value ^9^. Rosa *et al*. assessed diffuse interstitial myocardial fibrosis measured by T1 mapping in LFLG AS patients with and without FR in 41 LFLG AS and 24 high-gradient AS patients ^9^. The authors showed that indexed ECV was higher in patients with LFLG AS with and without FR in comparison with high-gradient AS (35.25 ± 9.75 versus 32.93 ± 11.00 versus 21.19 ± 6.47 mL/m ^2^, respectively; *P* <0.001). The indexed ECV and ECV levels were comparable in LFLG AS patients with and without FR ( *P* = 0.950 and *P* = 0.701, respectively). Rosa *et al*. concluded that the degree of fibrosis is the same in patients with and without FR, suggesting that myocardial fibrosis may not explain the lack of FR in patients with LFLG AS.

Although T1 mapping allows quantification of myocardial fibrosis, in dilated cardiomyopathy, there is a relative scarcity of studies comparing ECV and T1 with concurrent histological examination. Nakamori *et al*. examined the histological correlation of native T1 and ECV measurement for assessing myocardial fibrosis in dilated cardiomyopathy in 36 patients ^10^. T1 and ECV both demonstrated significant correlation with the biopsy-proven collagen volume fraction (r = 0.77 and r = 0.66, respectively; *P* <0.05). In addition, ECV showed a substantial association with the biopsy extracellular space component (r = 0.86). Nakamori *et al*. finally stated that T1 and ECV had comparable efficacy in measuring histological collagen volume fraction in dilated cardiomyopathy.

Although a number of metrics exist in CMR for measuring myocardial fibrosis, only limited data for stratification purposes are available ^11^. Treibel *et al*. evaluated the relationship between clinical outcomes and various indices such as ECV, native T1, post-contrast T1, and the partition coefficient in 1714 consecutive patients without amyloidosis or hypertrophic cardiomyopathy ^11^. The authors showed ECV demonstrating highest log-rank statistics and best separation of Kaplan–Meier curves. Furthermore, ECV was strongly linked to clinical outcomes in univariate and multivariate analyses. Treibel *et al*. stated that ECV measurement of myocardial fibrosis was closely associated with outcomes in relation to other CMR metrics.

## Limitations of T1 mapping

Although T1 mapping has clearly shown its potential for quantitative tissue characterization, there are still issues that need to be overcome for widespread acceptance ^3, 7^. There is no consensus in the CMR community’s methodology and approach ^7^. Some form of standardization along with histological validation is required for universal implementation. The absence of reference values for normal and abnormal myocardium for vendor-specific sequences hinders its approval ^12^. T1 mapping does not have any inbuilt features for heart rate modification ^12^. Furthermore, standardization of the acquisition phase is needed as it can affect T1 and ECV values because of the oscillating myocardial blood volume ^12^.

## T2 mapping

T2 mapping is a CMR approach used to generate a parametric image or map from a series of input images and calculations ^13^. As a result, the created map mirrors calculated T2 relaxation time at each pixel. This can be performed in any cardiac slice and position ^13^. T2 mapping holds considerable promise for more precise inflammation/edema imaging compared with traditional T2-weighted imaging. Additionally, T2* mapping can be used for quantification of myocardial siderosis in disease states that can lead to myocardial iron overload.

Acute inflammatory cardiomyopathy is a cardiovascular condition that requires biopsy to detect disease activity. Some studies have suggested that T2 mapping may become an integral tool to facilitate evaluation of inflammation within the myocardium non-invasively ^13^. There is growing evidence that suggests that T2 mapping may be more indicative for acute inflammation than T1 mapping ^14^. T2 mapping is also sensitive to identification of water in a more chronic setting such as scarring or ischemia ^14^. Interestingly, in the MyoRacer trial, T2 mapping was the only CMR parameter with acceptable diagnostic accuracy (73%) for recognizing biopsy-proven myocarditis for patients with chronic symptoms longer than 14 days ^15^.

## Strain imaging

Evaluating cardiac function has always been complex or cumbersome in cardiology and this applies particularly to ejection fraction ^4^. Although ejection fraction is the conventionally used metric to assess cardiac function, there are a number of limitations to be acknowledged ^4^. This can be attributed to the suboptimal reproducibility, volumetric nature, and inability to show regional left ventricular function ^16^. Strain imaging is an emerging modality which provides direct information on myocardial deformation. Furthermore, myocardial strain is a marker of left ventricular health and mechanics beyond left ventricular ejection fraction, and it measures the transition from a relaxed to a contractile state ^17^. This type of imaging may be helpful to detect preclinical left ventricular dysfunction prior to decreases in the left ventricular ejection fraction.

## Cardiovascular magnetic resonance tagging

CMR tagging is an approach that allows visualization of transmural myocardial involvement without using physical markers ^18^. There are a number of available tagging sequences, including spatial modulation of magnetization (SPAMM), delay alternating with nutations for tailored excitation (DANTE), complementary SPAMM (CSPAMM), harmonic phase (HARP), displacement encoding with stimulated echoes (DENSE), and strain encoding (SENC) ^18^. Each of these methods has different versions for improved resolution, signal-to-noise ratio (SNR), scan time, anatomic coverage, image quality, and three-dimensional (3D) capabilities ^18^. Unlike feature tracking (FT) strain, they require a separate dedicated sequence and post processing.

CMR tagging is a commonly accepted reference standard for strain quantification ^4^. The process labels different areas of the myocardium with unique radiofrequency saturation planes to create dark lines ^19^. Subsequently, a tagging formation is created which forms a grid of markers known as tags. As a result, tracking these tags facilitates visualization of myocardial deformation or strain. Tagging is inherently affected by the magnetic properties of the tissue.

There are also a number of limitations of CMR tagging ^4^. For instance, images with tags may have low temporal resolution. Strain may be underestimated if the tag does not correspond to the initiation of cardiac contraction. Strain values may be less precise if measured at the endocardial border or thin-walled areas of the left ventricle. Lastly, special software is necessary for strain and it is a laborious process.

## Feature tracking

FT in CMR is a post processing technique that can be applied to standardly acquired cine steady-state free precession (SSFP) images. This method does not require a dedicated separately acquired sequence or intricate post processing ^20^. There are differences between FT and CMR tagging ( Table 1). It detects anatomic characteristics in the myocardial borders in the CMR image and identifies areas of interest within these regions. These areas are tracked in the cardiac cycle by looking for similar areas in the subsequent images. There are benefits and disadvantages to FT ^4^. The strain in FT can be used with a number of different software programs. FT strain tracks movement of in-plane points within the myocardium as opposed to some of the other dedicated CMR strain acquisitions, which track changes of the myocardium within each voxel or pixel.

**Table 1.** Differences between cardiovascular magnetic resonance tagging and feature tracking strain.

	Cardiovascular magnetic resonance tagging	Feature tracking strain
Temporal resolution	Low	Low
Spatial resolution	Low	Low
Commercial software	Many	Few
Post processing time	Long	Small
Validation studies	Many	Few
Image acquisition time	Long	No additional image acquisition time
Image analysis	Can be difficult, requires special software	Not difficult
Reproducibility	High	Good
Types of strains generally performed	Strain can be performed in all directions (2D and 3D)	Longitudinal strain, circumferential strain, and radial strain can be performed

## Recent studies using strain in cardiovascular magnetic resonance

Gatti *et al*. explored the role of FT strain in CMR for detecting subclinical systolic and diastolic dysfunction in 30 acute myocarditis patients with preserved ejection fraction ^21^. In addition, 24 normal patients served as controls. The inter-observer variability in the 2D and 3D FT CMR had a *P* value greater than 0.42, intra-class correlation greater than 0.80, and n ^2^ greater than 0.98. Interestingly, the inter-observer variability in global, radial, circumferential, and longitudinal strain showed no statistical difference between systolic and diastolic strain rate ( *P* = not significant).

Romano *et al*. investigated the relationship of FT global longitudinal strain (GLS) during vasolidator stress CMR with major adverse cardiac events (MACEs) in 535 patients with a follow-up of 1.5 years ^22^. Previous studies have indicated that blunted myocardial strain may be linked to an adverse prognosis during dobutamine stress echocardiography. Patients with stress GLS equal to or greater than the median had greatly decreased event-free survival compared with stress GLS of less than the mean (log rank *P* <0.0001) on Kaplan–Meier analysis. Furthermore, the stress GLS was substantially linked with MACE after adjustment for clinical and imaging variables (hazard ratio = 1.267, *P* <0.0001). Romano *et al*. concluded that FT GLS was an independent predictor of MACE during vasodilator stress CMR in patients with coronary artery disease.

Leng *et al*. examined the significance of right atrial (RA) dysfunction on pulmonary arterial hypertension (PAH) by using RA longitudinal strain in 80 PAH patients and 80 normal patients with CMR ^23^. Compensated and decompensated states have been known to affect survival in patients with PAH. RA strain correlated with elevated RA pressure (r = −0.57; *P* <0.0001), right ventricular volume (r = −0.37; *P* = 0.002), biomarkers (r = −0.53; *P* <0.0001), and lower exercise capacity (r = 0.41; *P* <0.00001). In addition, RA passive strain was seen as the best predictor of composite adverse events (C statistic = 0.75, hazard ratio = 0.84; *P* = 0.019). Leng *et al*. concluded that RA strains were associated with right ventricular decompensation and elevated risk of adverse events.

## Limitations of strain in cardiovascular magnetic resonance

Strain is largely used in the research setting, and a number of issues need to be resolved for successful integration into clinical practice ^4^. For any diagnostic or clinical test to be implemented, a normal range of values needs to be established ^4^. Values for method and software cutoff need to be established. There is no validation against a universal reference standard. Each method and software must undergo rigorous testing before adoption into clinical practice. In general, data suggest that GLS should be used for assessing global ejection fraction rather than segmental strain, and validation and inter-vendor agreement are of less concern with GLS in comparison with other types of strain values ^4^.

Furthermore, values from speckle tracking echocardiography may not always correlate with values from CMR strain. Other clinical factors such as blood pressure, heart rate, age, and gender may also affect strain values ^24^. Strain at baseline and follow-up needs to be measured by the same software and analysis for reproducible values.

## Artificial intelligence

Machine learning (ML), a subset of AI, has allowed the field of cardiology to escape the confines of conventional inquiry and embark on a new realm of multi-dimensional information occurring in real time leading to data discovery–driven research ^25, 26^. First and foremost, coronary artery disease is the most common cause of death in the developed world ^27^. ML is not a distant concept on the horizon but an inevitable necessity in the evolutionary line of cardiovascular imaging and clinical care, especially in CMR. AI is exponentially expanding every sector of human information, from self-driving cars to speech recognition software ^28^. The massive influx of large data emanating from wearable devices, medical apps, electronic health system, and imaging systems will supersede the capabilities of existing statistical software ^25^. Nevertheless, ML can unravel information present within this vast data matrix to dramatically enhance disease prognostications and survival predictions ^29^.

## Types of machine learning

ML is an umbrella term used to encompass a variety of algorithms ^30, 31^. Two of the most frequently used ML algorithms are supervised learning and unsupervised learning ( Table 2). Supervised learning uses a dataset labeled with classes or outcomes ^30, 31^. Unsupervised learning can be considered agnostic; it works with datasets without any labels or classification to unravel hidden relationships ^30, 31^. Semi-supervised learning, a hybrid of the earlier-mentioned approach, uses both labeled and unlabeled outcomes within a dataset to discover relationships ^30, 31^. Reinforcement learning is similar to human psychology, using reward criteria within a dataset ^30, 31^. Reinforcement learning currently has a limited role in CMR and cardiology.

**Table 2.** Types of machine learning.

Machine learning algorithms	Description	Types
Supervised learning ^30, 31^	Dataset contains labels and outcomes	This includes logistic regression, Bayesian network, random forests, elastic net regression, and least absolute shrinkage and selection operator (LASSO) regression.
Unsupervised learning ^30, 31^	The algorithm deciphers relationships in datasets without labels.	This includes K-means clustering, hierarchical clustering, and principal component analysis.
Semi-supervised learning ^30, 31^	Dataset contains labeled and unlabeled classes and outcomes.	It is a mixture of supervised and unsupervised learning, used in speech and image recognition.
Re-enforcement learning ^30, 31^	Similar to psychology, uses reward function	Based on human psychology. Used in analytics, imaging, and disease screening

 Deep learning is gaining massive momentum as it has unlimited potential ^26, 29^. The deep learning framework has an architecture similar to that of the human brain; it uses multiple layers like neuronal networks. Furthermore, deep learning is expanding because of explosive growth in cloud infrastructures and computing capabilities in current technology.

## Recent studies in artificial intelligence for cardiovascular magnetic resonance

A number of centers have explored the potential of ML in CMR recently. Winther *et al*. used a deep learning algorithm for automatic segmentation of the right and left ventricular endocardium and epicardium to measure cardiac mass and function ^32^. Image segmentation can be time-consuming and particularly challenging in CMR. This was applied to a number of datasets, which included the Hannover Medical School data science bowl cardiac challenge and the Medical Image Computing and Computer Assisted Intervention (MICCAI) 2009 left ventricular segmentation challenge. Interestingly, the deep learning approach accomplished outcomes comparable to or greater than those of human experts. These findings must be taken with a degree of caution because of the small sample sizes.

Tan *et al*. explored the role of a convolutional network, a deep learning approach, for automatic segmentation of the left ventricle in all short-axis slices ^33^. This ML approach was applied to a number of publicly available datasets, which included the left ventricular segmentation challenge dataset containing 200 CMR imaging sets with diverse cardiac pathology. Surprisingly, the authors obtained a Jaccard index of 0.77 in the left ventricular segmentation challenge dataset. In addition, they obtained a continuous ranked probability score of 0.0124 with the Kaggle second annual data science bowl. As a result, Tan *et al*. showed the potential of the ML algorithm in automatic left ventricular segmentation in CMR.

Bai *et al*. used a fully convolutional network for automated analysis of CMR images from a large imaging database consisting of 93,500 images in 5000 patients for calculating left and right ventricular mass and volumes ^34^. On a short-axis image test of 600 patients, the Dice metric measured 0.94 for left ventricular cavity, 0.88 for left ventricular myocardium, and 0.90 for right ventricular cavity. Furthermore, the average Dice metric measured 0.93 for the left atrial cavity in the two-chamber view, 0.95 for left atrial cavity in the four-chamber view, and 0.96 in RA cavity in the two-chamber view. Bai *et al*. showed that ML automated methods produce values comparable to those of human experts.

## Limitations of machine learning

ML will become an inevitable necessity as the number of medical apps, wearable devices, and miniaturized devices continue to grow and prosper ^25^. For successful integration into the clinical environment, a number of issues need to be addressed ^5^. ML algorithms require extensive exposure to large datasets to gain accuracy, and obtaining such datasets can be difficult ^26, 29^. Data sharing among institutions can be difficult and laborious because of multiple institutional review boards, and datasets ideally should be publicly available.

A universal standard is required for data standardization ^26, 29^. Although digital imaging and communications in medicine (DICOM) and picture archiving and communication system (PACS) are valuable for imaging data, there are differences in these programs between centers. There are a number of different classifications, protocols, and acquisition protocols among various institutions, and imaging and clinical data exist on separate user interfaces. As data become larger and more complex, manual data entry will become difficult. If newer software can successfully integrate clinical and imaging information, it can facilitate the expansion and utilization of ML in various academic centers.

## Conclusions

Although echocardiography is used primarily as the first-line imaging test for a majority of cardiovascular pathology, the field of CMR imaging has been steadily growing in recent years. CMR provides valuable information on myocardial tissue and function. Recent developments in T1/T2 mapping, strain imaging, and AI show considerable promise in CMR as they can greatly improve diagnosis and patient welfare. Nevertheless, these options still require further validation before full integration into clinical care. As technology continues to grow and improve, the future of CMR imaging will be very bright in the years to come.
